# Color Holography for the Documentation and Dissemination of Cultural Heritage: OptoClones^TM^ from Four Museums in Two Countries

**DOI:** 10.3390/jimaging5060059

**Published:** 2019-06-15

**Authors:** Andreas Sarakinos, Alkis Lembessis

**Affiliations:** Hellenic Institute of Holography, 28 Dionysou Street, 15234 Chalandri, Greece

**Keywords:** color holography, color holograms, cultural heritage, museum display

## Abstract

True-color holograms, as they are the most advanced and realistic three-dimensional images obtainable with current technologies, can become valuable tools for the preservation, documentation and diffusion of cultural heritage. In this respect, the transportable Z3^RGB^ color holography system and the HoLoFoS^TM^ illuminant developed by the Hellenic Institute of Holography have been successfully utilized for the in-situ recording and displaying of OptoClones^TM^ (Denisyuk-type color holograms) in four museums and two countries. The holographic image of an OptoClone^TM^ is characterized by a wide angle of view, full parallax and perspective, good color rendition and ultra-realistic reproduction of the optical properties of the materials of an artefact. In this paper, we report on our accumulated expertise in on-site holographic documentation of museum artworks of various types, already from four museums of world caliber and reputation (Athens and Thessaloniki Byzantine, Fabergé Museum of St. Petersburg and Diamond Fund of Russia). In one case, a world’s first, the in-situ recorded OptoClones^TM^ have been subsequently displayed as part of the permanent exhibition of the Byzantine & Christian Museum of Athens in replacement of the original artifacts while on loan. On another occasion involving State Treasures from the Diamond Fund of Russia, the recorded OptoClones^TM^ exhibited inside the Moscow Kremlin were highly appraised by officials and international experts as well as the general public allowing reasonable optimism for the prospects of Display Holography for museums.

## 1. Introduction

The Hellenic Institute of Holography (HiH) was established in Athens, Greece, in 1987 with the purpose of introducing and promoting holography in Greece in all possible areas: science, art, media and authentication. HIH has the status of non-profit scientific and educational organization with its income derived by members’ contributions and services to third parties.

Being active in a country with a unique cultural tradition of worldwide influence extending from Classical Ancient Greece to Orthodox Byzantium Christianity, the use of display holography in the preservation, recording and public visual dissemination of artifact items for cultural heritage has been at the core and forefront of the activities of the HiH.

In this framework during the period of January 1995 till the end of April 1996 HiH participated in the project “Holography applied to protection, preservation and non-destructive evaluation of cultural heritage” funded by EU IC-INTAS program. The program was coordinated by the late Prof. Pierre Boone of Universiteit Gent with participants the National Academy of Sciences of Ukraine (Prof. Vladimir Markov), the Russian Academy of Sciences (Prof. Yuri Denisyuk) and HiH. An excerpt from the project’s objective:

“Holography offers extraordinary potential applications in the domain of cultural heritage. Two main trends exist. One is that of display holography, where holograms are used as a means of visual information representation, for example, to exhibit optical copies of museum items. The second direction is connected with technical applications of holography and can be used to solve problems related to the study, conservation and restoration of museum items, e.g., by checking the present state of integrity of objects and locating and characterizing deterioration before it becomes critical.” [1].

More than ten years later (2009), on the grounds of technological progress in various disciplines such as solid-state lasers, panchromatic emulsions, LED illumination etc., the HIH set out its own HoloCultura program for the use of color display holography in applications related to cultural heritage.

During Phase I of this program, the necessary (trans) portable equipment, processes and software for the in situ recording of artifacts and their subsequent interpretation in either analog or digital display holograms were developed. Considering security problems, it was clear that mobile recording equipment was necessary to make display holography accepted as an advanced optical documenting technique to be performed inside museum buildings. In Phase II experimental work was done by using different materials for producing color display holograms of acceptable standard by the prospective end-users using our in house developed systems and processes.

During Phase III (2013 onward) application projects in the area of color display holography for cultural heritage were successfully conducted by recording a large number of OptoClones^TM^ in four museums in two countries under non laboratory conditions. The success of Phase III was sealed by two Int’l Association of Hologram Manufacturers (IHMA) awards in 2015 and 2017.

## 2. OptoClones Technical Considerations

In theory, true color Denisyuk-type holograms of high quality can be recorded in silver halide emulsions and subsequently be displayed reproducing ultra-realistic three-dimensional images [2], if certain prerequisites are fulfilled [3,4,5,6] e.g.,:

- Suitable selection of three or more laser wavelengths
- Panchromatic recording plates with extremely low scattering
- Optimized processing of the exposed plates
- Suitable recording geometry to eliminate dispersion
- Mechanical and thermal stability during recording
- Optimized illumination of the color hologram to enhance depth reconstruction, color rendition and minimize blur.

Moreover the design of a holographic camera for the recording of high quality color holograms inside a museum introduces more challenges to the overall optical and mechanical design of such a system.

Based on the above prerequisites, HiH developed during Phase I of the HoloCultura program the Z3^RGB^ color holography transportable system and the HoLoFoS^TM^ RGB led illuminating devices for the in situ recording and subsequent display of high-quality color Denisyuk-type holograms as reported in our papers [7,8].

In a holistic approach to color holography for cultural heritage applications, from the very beginning, we had considered that an enhanced illuminating device is an indispensable pair to a high quality color hologram. The tuned pairs of our color holograms and the HolofoS^TM^ illuminating devices we have termed as OptoClones^TM^ not only because the holographic image reconstructed from such pairs is ultra-high realistic but also to distinguish from the incorrect use of the word “hologram” in contemporary so called “hologram” applications.

### 2.1. The Z3^RGB^ Color Holography Recording System

**Figure 1:**
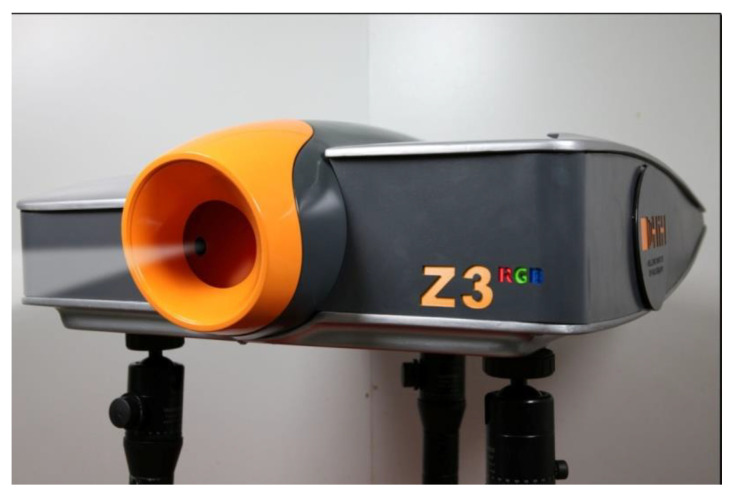
The ZZZyclops color holography camera (the white beam is emulated) (© 2013 HiH).

**Figure 2:**
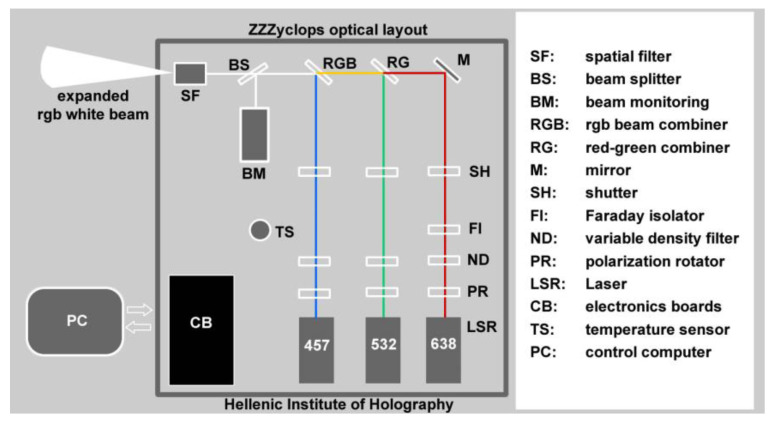
Inner layout of the ZZZyclops color holography camera.

The Z3^RGB^ system’s color holography camera model ZZZyclops (Figure 1 and Figure 2) currently incorporates two DPSS lasers (Cobolt lasers) and one stabilized DL system (HiH) with TEM00 emissions and coherence lengths of more than five meters each. The three lasers are:

- Red laser: 638 nm at output power 90 mW (HiH-R100 DL laser);
- Green laser: 532 nm at output power 100 mW (Cobolt Samba DPSS laser);
- Blue laser: 457 nm at output power 50 mW (Colbolt Twist DPSS laser).

The HiH-R100DL laser system was assembled by HiH engineers and added to the camera in mid 2013 as a replacement for the red laser originally fitted in 2012. This system comprises of a diode laser TEC housing, a high accuracy TEC and diode laser current driver, a selected single mode red diode laser and a Faraday optical isolator.

A further improvement to the original system was the addition of two more electro-mechanical shutters in 2015 to control the exposure times for each laser separately.

The red, green and blue laser beams are mixed coaxially into a “white” light beam. This mixed RGB beam is used to illuminate the holographic plate and the object during hologram recording.

The camera is controlled by software developed in-house. The software runs on a personal computer (PC) interfaced to the ZZZyclpos’ analogue to digital and digital to analogue boards (CB) through a USB bus. The camera incorporates a scanning Fabry–Pérot interferometer (BM) to monitor the single line and mode hop free operation of the three lasers. The software controls the electro mechanical shutters (SH) and provides a real time graphical view of the BM output.

The lasers and optics are mounted on a 60 by 60 cm honeycomb optical breadboard which in turn is enclosed in a fiber glass protective housing. The housing sits on a pneumatic carrier allowing height and tilt adjustments.

A two layered vibration isolation platform has been developed on which the object and the holographic plate are placed during the recording stage. The Z3^RGB^ system includes a special foldable and light-proof exposure chamber (tent) to enclose the plate/object space. A transportable darkroom tent completes the system and may be used in cases where there is no available photographic darkroom in the premises.

### 2.2. The HoLoFos^TM^ RGB Color Hologram Illuminant

**Figure 3:**
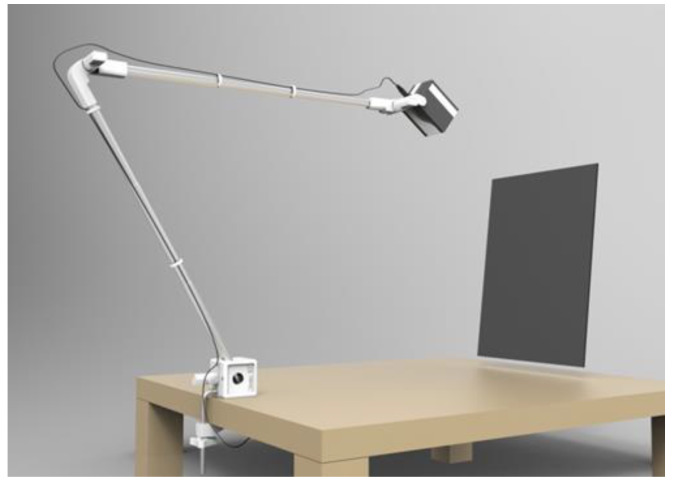
The latest generation of HoLoFoS^TM^ model (λ).

**Figure 4:**
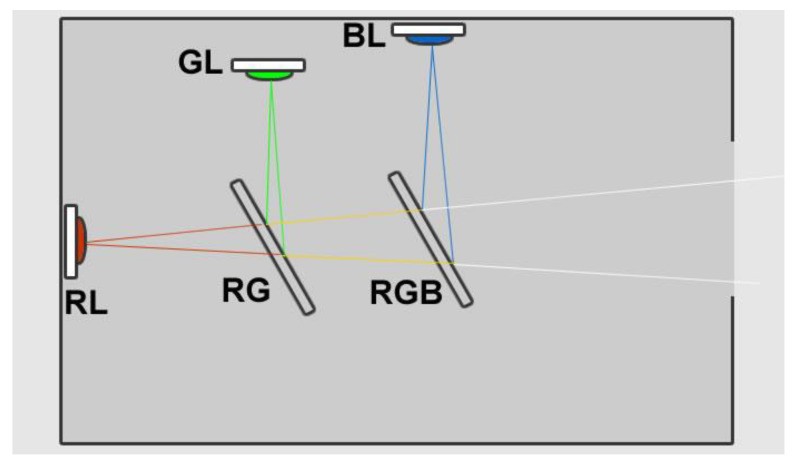
HoLoFoS^TM^ optics head layout. RL, GL, BL: red, green, blue LEDs. RG: red-green combiner. RGB: red-green-blue combiner.

**Figure 5:**
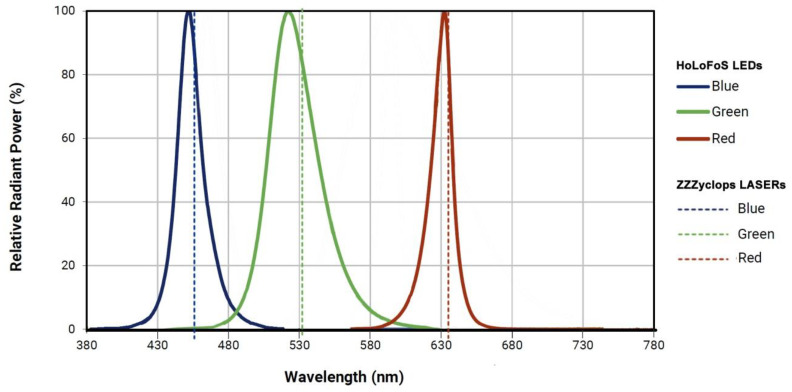
LEDs spectrums of the HoLoFoS^TM^ device and emission lines of the lasers incorporated in ZZZyclops.

The HoLoFoS^TM^ (Figure 3) is an LED special illuminating device emitting narrow bandwidth red, green and blue light with intensity peaks near the recording wavelengths of the ZΖΖyclops color holography camera. The HoLoFoS^TM^ provides coaxial mixing of the RGB LEDs beams into a white light beam with the use of dichroic combiners (Figure 4). The close matching of the HoLoFoS^ΤΜ^ LEDs wavelength bands to the recording wavelengths of the ZZZyclops camera (Figure 5) ensures the proper color reproduction of reflection holograms recorded with the camera.

Typical bandwidths of the LEDs incorporated in the device at FWHM are 15 nm for the red, 30 nm for the green and 16 nm for the blue.

At the same time, the small footprint of the HoLoFoS^TM^ LED sources (2 mm by 2 mm per color) allows placing the HoLoFoS^TM^ at a closer distance to a reflection type hologram while retaining image sharpness and enhanced depth. Electronic control of the LEDs driving currents permits the accurate intensity mixing of the emitted red, green and blue bands in order to fine tune the colors of the reconstructed images.

The latest HoLoFoS^TM^ model (λ) has a redesigned telescopic mounting arm plus a redesigned optics head with better quality dichroic combiners and more powerful LEDs than previous models.

## 3. Recording OptoClones^TM^ Inside Museums

**Figure 6:**
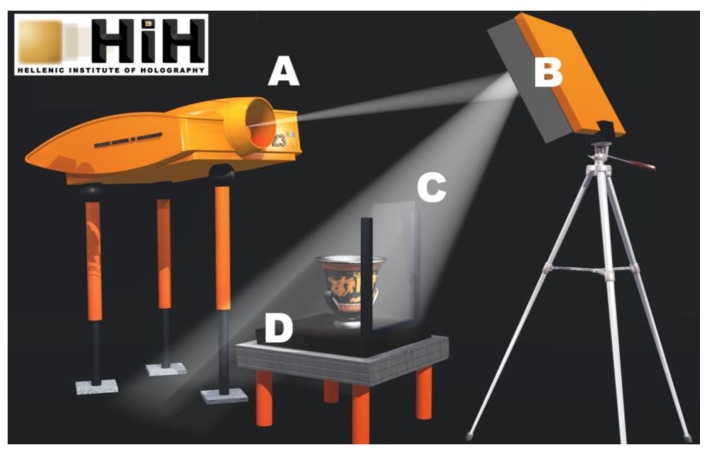
The mixed RGB laser beam from the ZZZyclops camera (A) is steered by mirror (B) to the holographic plate (C) and the object placed on the vibration isolation platform (D). The light reflected from the object interferes with the beam on the plate’s plane. The plate records the interference pattern during exposure.

In the years 2013–2017, the HiH produced OptoClones^TM^ for the documentation of artworks and display purposes in collaboration with museums in Greece and Russia. These projects were carried out during the museum’s working hours and under strict security measures in rooms with varying temperature and humidity conditions. After installing the Z3^RGB^ system camera in the room specified by the museum officials a number of preliminary test recordings were run to familiarize the conservators with the holographic process. The recording principle is depicted in Figure 6.

The artifacts to be recorded in a session were brought in the room 30 min to 1 h before recording to ensure temperature and humidity equilibrium, and to relieve any mechanical stresses induced by their transfer. Due to museums’ security policies two or more of the museum personnel (conservators and security) were always present in the room. The established procedure for production recordings were:

1. Place the object to be recorded in a special exposure box lying on the anti vibration platform inside the lightproof exposure chamber (normal light).
2. Establish exposure parameters and recording geometry (safety light).
3. Place a holographic photosensitive plate of suitable dimensions on the front of the exposing box (safety light).
4. Lett the plate stabilize (10 min) (lights off).
5. Expose the plate to the combined reference and object light waves (typically 15–25 s for a 30 × 40 plate) (laser exposure light).
6. Remove the exposed plate (safety light).
7. Repeat steps 3–5 depending on the number of copies to be recorded.
8. Remove the object from the exposure box (normal light).

Steps 1 and 8 were always carried out by a conservator, steps 3 and 6 either by a conservator or the first author of this paper, steps 2 and 5 by first author of this paper.

Typical distance of object to plate was in the range of 5–10 mm. The mean energy dosage on the holographic plates was 120–200 μJ/cm^2^ per color (red, green, blue) depending on the surface characteristics of the object.

Following exposure, each plate underwent wet chemical processing. Typical stages of the wet processing were:

- Chemical development in a developer tuned to the holographic plate (safety light).
- Rinse with distilled water (safety light).
- Bleaching (safety light).
- Short rinse with distilled water (normal light).
- Prolonged rinse with tap water (normal light).
- Short rinse with distilled water (normal light).
- Short bath in a solution of a few drops of a wetting agent (Photo-Flo) in distilled water (normal light).
- Letting the plate to dry naturally (HoLofoS^TM^ light to monitor the drying process and quality inspection).

Depending on the photosensitive plates’ origination a suitable developer and bleach were used (Table 1).

The wet processing is a critical procedure and has to be carried out by a person skilled in the art. If not carefully executed it may introduce distortions and color shifting to the final holographic image. In all projects the wet processing was carried out by a HiH expert (either the first author of this paper or HiH assistant holographer K. Sarakinos). Using these procedures we were able to record, fully process and quality control an OptoClone^TM^ typically within 30 min.

### 3.1. Optolones^TM^ in the Byzantine And Christian Museum (BXM) Athens, Greece (2013)

In August 2013 the Byzantine & Christian Museum of Athens cooperated with HiH in order to document as OptoClones^TM^ twelve selected objects from its collections that were to travel abroad as a loan to the temporary exhibition “Heaven and Earth: Art of Byzantium from Greek Collections” (U.S.A October 2013–August 2014) plus a pilgrim token of 6th c.AD “Eulogy of St. Mamas” that was scheduled to be displayed in the Thessaloniki Museum of Byzantine Culture in Oct. 2013 (Table 2).

**Figure 7:**
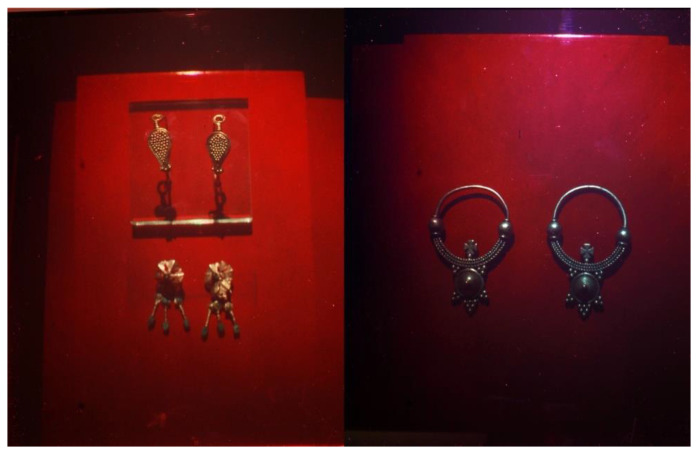
Left: Photograph of the OptoClone-BXM1, two pairs of golden earrings, 4th c AD (© 2013 HiH). Right: Photograph of the OptoClone-BXM6, silver earrings, 4th c. AD (© 2013 HiH).

The ZZZyclops camera was installed in BXM’s ground level in a room of the micro ceramics restoration lab. After exposure each plate was transferred in a light proof box to a small darkroom on the second floor of the building for chemical development, drying, and quality inspection. The BXM OptoClones^TM^ (Figure 7) were recorded on Ultimate U08 plates by the first author of this paper.

The OptoClone-BXM7 depicts both the front and back faces of the object in a side by side double exposure on the same plate. The OptoClones^TM^ (BXM1 to BXM6) were displayed in place of the original objects for the period that the original objects were on loan.

For the first time in Greece–but equally a world’ first–full-color holograms recorded in situ with the use of proprietary portable equipment had been exhibited as part of the permanent collection of a Museum in the place of original objects for the period that these were on loan abroad.

As stated by Nikos Konstantios (Archaeologist, Museologist. Byzantine & Christian Museum, Athens): “We have opted to use display holograms—instead of digital media–for the visual replacement of selected cultural artifacts during their temporary loan as we felt that their one-to-one ultra-realistic 3D optical representation through full-color holography allows the viewer to form an accurate view of the object–even when the original artifact is not present. Moreover, this happens instantly at first glance without any interaction or complications introduced by the digital media (touch screens, buttons, image quality etc.)”.

**Figure 8:**
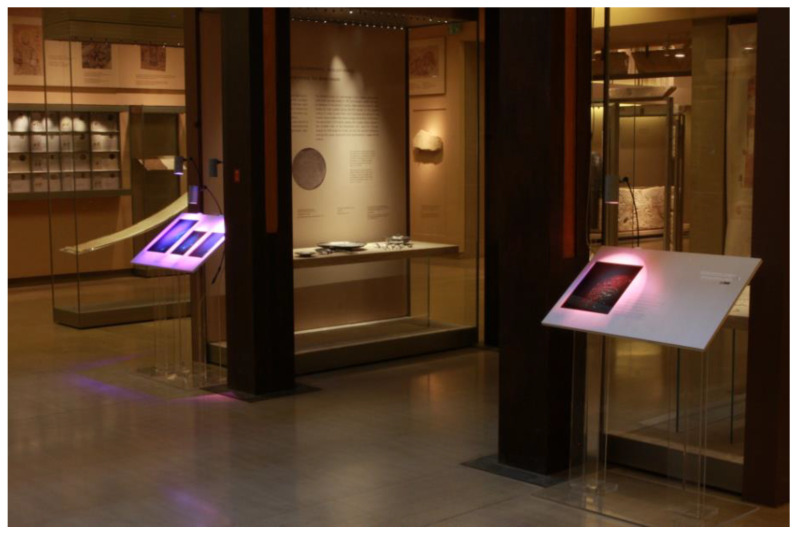
Display stands with OptoClones^TM^ and HoLoFoS^TM^ illumination, Byzantine and Christian Museum of Athens (BXM), June 2014 (HiH, 2014).

It is worth noting that, for the chromatically correct and realistic replay of the holograms, the HoLoFoS^TM^ RGB illumination systems were used. The OptoClones^TM^ and the HoLoFoS^TM^ illuminants were incorporated by Yanni Georgaras (Red Dot awarded Industrial Designer) into custom display stands in cooperation with the relevant services of the Museum (Figure 8).

### 3.2. Optoclones^TM^ in the Museum of Byzantine Culture (MBC) Thessaloniki, Greece (2013)

**Figure 9:**
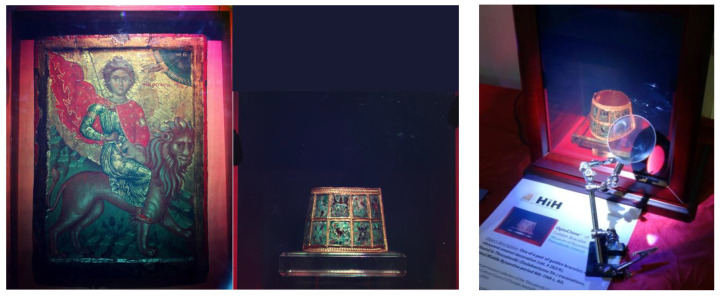
**Left**: Photograph of the OptoClone^TM^ of the Saint Mamas icon (© 2013 HiH). **Center** and **Right**: Two views of the same OptoClone^TM^ of one of a pair of bracelets MBC cat. BKO262/6, 10th c. AD (© 2013 HiH).

In September 2013, using the Z3^RGB^ transportable system, the HiH in collaboration with Thessaloniki Museum of Byzantine Culture recorded OptoClones^TM^ of the Saint Mamas icon and selected objects of the museum’s collections for display purposes and documentation before and after conservation (Figure 9).

The holy icon of St. Mamas had been transported from its origin in Cyprus to Thessaloniki for conservation and subsequent display during the exhibition “Veneration of Saint Mamas in the Mediterranean: A Traveler Border–Defender Saint” (MBC, October 2013 to January 2014). The recorded in situ OptoClone^TM^ of St. Mamas and the previously recorded in BXM Athens “St. Mamas Eulogy” (OptoClone–BXM7, Table 1) were also displayed enhancing the entrance of the exhibition hall.

The official inauguration of the exhibition was declared by his His Most Divine All-Holiness the Archbishop of Constantinople, New Rome, and Ecumenical Patriarch of Eastern Orthodox Church.

The HiH mobile equipment was sent from Athens to Thessaloniki and installed in the photography lab of the museum. The adjacent small darkroom was used for the chemical processing of the plates. The MBC OptoClones^TM^ were recorded on Ultimate U08 plates by the first author of this paper.

### 3.3. Optoclones^TM^ of the Fabergé Imperial Eggs, Museum Fabergé Saint Petersburg (2015)

The Fabergé Museum opened to public for the first time in November 2013 dedicated exclusively to the artistic genius of Carl Gustav Fabergé. The Museum collection has at its epicenter nine of the world-famous Fabergé Imperial Eggs (previously known as part of the Forbes Collection).

The signing of an agreement between the Fabergé Museum at the end of 2014 with the support of ITMO University of St. Petersburg was the starting point for the production of OptoClones^TM^ of the nine Imperial Eggs, three Eggs of Imperial Quality plus an additional Fabergé piece of art “Desktop Timepiece with a Globe” (Table 3).

In the spring of 2015 the mobile equipment of HiH was sent from Greece to St. Petersburg where it was installed in the premises of the Museum. The room assigned to HiH was the high-security metals cleaning lab in the basement of the museum, heavily guarded throughout the recording sessions. The same room served HiH as a darkroom for wet processing the plates. The Egg to be recorded was brought in the room by the assigned conservator accompanied by two security officers who would stay in the room until the end of the specific recording session, while another guard was on duty outside the double metal doors of the room.

**Figure 10:**
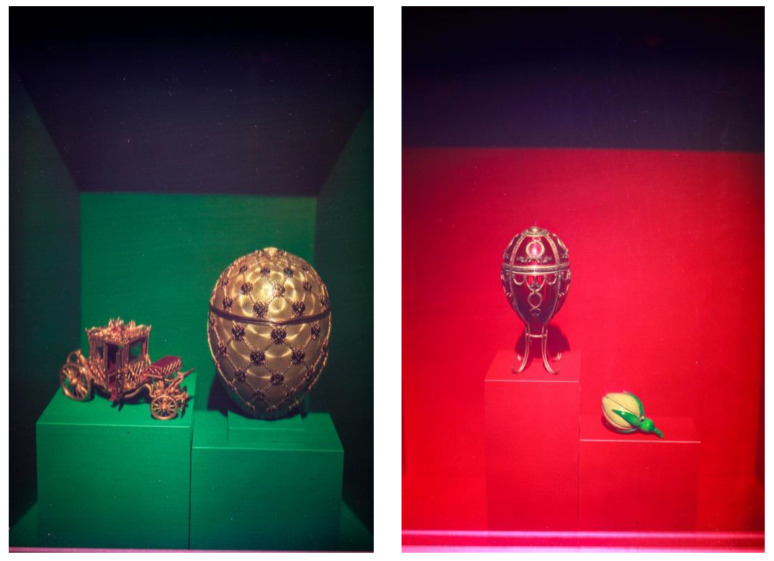
Photos of the recorded OptoClones^TM^ of the Coronation Imperial Easter Egg (**Left**) and the Rosebud Imperial Egg (**Right**) (© 2015 HiH).

Following a period of almost 2 months of daily involvement and more than 200 holographic shots in dark-room conditions, five almost identical collection sets of unique holographic optical clones of 13 selected artworks of the Museum were delivered (Figure 10). These OptoClones of 30 × 40 cm size were recorded by the first author of this paper on BBpan plates manufactured by Color Holographic (UK) and supplied to HiH under exclusive non-commercial license.

**Figure 11:**
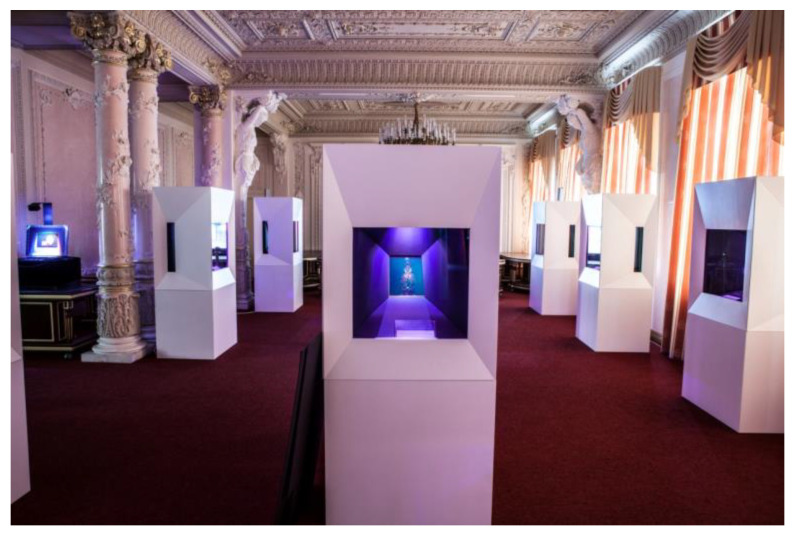
The display cases with the Fabergé Egg OptoClones^TM^ at the Magic of Light Exhibition, ITMO University 2015 (HiH, 2015).

The collection was first exhibited in the exhibition “Magic of Light” in Saint Petersburg, Russia (June–October 2015) hosted by ITMO University and co-organized by HiH. Special display cases incorporating HoLoFoS^TM^ devices were built to enhance these exhibits (Figure 11).

For this collection HiH received the IHMA “Excellence in Holography-Best of Year 2015” [9] award, which is not conferred annually unless there is an outstanding project fulfilling the high standards set by the judges. It must be noted that—apart from the production of these unique holograms—the award also refers to the way the necessary aspects of suitable presentation and illumination have been addressed in a concerted manner.

### 3.4. Optoclones^TM^ of Russian State Treasures Kremlin Diamond Fund, Moscow (2017)

In November 2017, for the celebration of the 50th Anniversary since the opening of the Diamond Fund, the Russian government (Ministry of Finance) decided to impress the public with the modern use of Yuri Denisyuk’s invention, that of reflection display holography.

The HiH, as part of its collaboration agreement with ITMO University of St. Petersburg, responded to the open public tender call and recorded 11 invaluable historical artworks officially designated as ‘State Treasures’ into holographic OptoClones^TM^ (Table 4).

It is worth noting that it may have been the first time ever when an open public tender was called for the in-situ creation of holographic optical clones to be delivered as finished products in a display setup along with the necessary associated illuminants, an interesting precedent in its own merit.

On account of the unique and invaluable nature of these artifacts, the project of their holographic documentation assumed the transport and installation of the Z3^RGB^ system inside controlled access areas and the in situ completion of all recording works.

**Figure 12:**
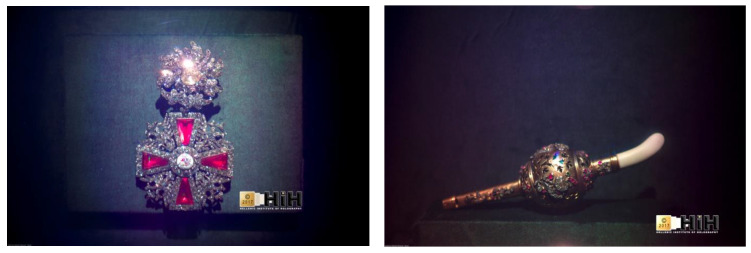
Photos of the recorded OptocClones^TM^ of Order of St. Alexander Nevsky (**Left**) and of the Imperial Whistle Cum Rattle (**Right**) (Photos by A. V. Evdokimov, 2017. © 2017 HiH).

The room assigned for the production was a conference room opening to a kitchenette with a double sink which was used as a darkroom for the chemical development of the plates. For almost one month during the summer of 2017, under strict monitoring conditions inside the heavily guarded high-security building of the State Treasury of the Russian Federation in Moscow, the holography team of HiH (A. Sarakinos, K.Sarakinos, A. Lembessis, M. Shevtsov) with the daily support of ITMO University, generated the new Gokhran Collection of OptoClones^TM^ (Figure 12).

Special attention was required for the objects ornate with hundreds of small precious stones. Recording single-beam reflection holograms of objects with highly specular surfaces on silver-halide plates is a demanding task requiring precise optimization of the exposure parameters and fine-tuning of the chemical processing. This requirement is valid even for conventional photographs (digital or film) of such artworks, but its significance is aggravated when it comes to three-dimensional depiction of this kind of object, such as jewelry.

A highly reflective plane facet of a precious stone or metal will reflect a large proportion of the reference beam back to the holographic plate resulting in recording a plane grating of a well-defined contour on a small area at the surface of the hologram. These high-intensity areas may ‘burn’ the emulsion if not properly exposed or create obscuring and abrupt changes of diffraction efficiency, especially in off-axis viewing relative to the diffuse holographic image of the facet.

**Figure 13:**
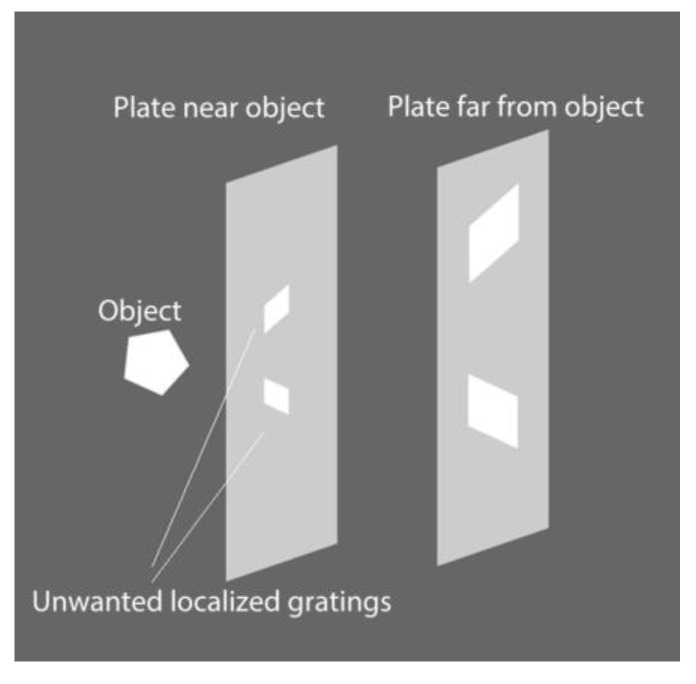
Specular reflections produce unwanted diffraction gratings obscuring the diffuse holographic image.

These effects are minimized when the facet is in close proximity to the holographic plate; in this case the plane grating coincides with the diffuse image of the facet and covers a minimum area on the hologram’s surface. A serious drawback of such a recording (i.e., the specular object being nearly in contact with the plate) is that high intensity radiation is localized in a very small area on the plate usually resulting in totally ‘burning out’ of the emulsion. These shortcomings are exponentially multiplied by the number of gemstones on the object and in cases of artworks decorated with a large number and variable types of precious stones, they become a serious obstacle for a successful holographic recording (Figure 13).

Therefore, in order to properly record holograms of the aforementioned type of artifacts, the holographer has to decide on the optimum distance of the object to the plate, on proper exposure to cover the allocation of dynamic range of intensities on the plate and on fine-tuning the developing chemistry and process to suppress the diffraction efficiency of the unwanted plane gratings.

In the Gokhran project, we carefully adjusted the object-to-plate distance so as to minimize the areas of the unwanted plane gratings while keeping the dynamic range of lasers radiation intensities and exposures within the dynamic range of the specific emulsion avoiding the ‘burning out’ of the emulsion. The plates of choice for this demanding project were the CH-BBpan (UK), which to our experience are characterized by a broad and consistent dynamic range. We used a fine-tuned compensating developer formula, adjusted the developing time accordingly, and introduced variable agitation to suppress the unwanted high-intensity plane gratings.

As a result, after several trials per object, we successfully recorded a series of full-color Denisyuk-type holograms of artifacts made of precious metals and adorned with multi-faceted precious stones; these OptoClones^TM^ under HoLoFoS illumination reconstruct holographic images without any plane gratings of the specular facets of gemstones obscuring the diffuse holographic image, even in off-axis-viewing.

These OptoClones^TM^ mostly in dimensions of 30 × 40 cm were recorded by the first author of this paper on BBpan plates made by Color Holographic (UK) and exclusively supplied to HiH under limited license for non-commercial use.

They were finish-sealed under the supervision of the first author of this paper in a second phase during September 2017 in the lab area of ITMO University in St. Petersburg. It was there where their framing and fitting of the proprietary HiH illuminants HoLoFoS-(λ) took place in preparation for their world premiere public presentation in a special event at the St. Andrews’ Hall of the Central Palace in Moscow Kremlin on November 16, 2017 with official guest, the Premier Minister of the Russian Federation Dmitry Medvedev.

On the same date, this project which sets new frontiers in display holography was presented at the annual international meeting The Holography Conference 2017, which took place in Barcelona, Spain. During the meeting the HiH received Commendation for ‘BEST DISPLAY APPLICATION OF HOLOGRAPHY’ at the annual IHMA Excellence-in-Holography Awards 2017 [10].

## 4. Conclusions

The potential of display holography for museum applications was examined in depth by researchers in the field for more than 30 years ago [11] but the wide use of holograms in museums never really happened. The main problems were the lack of suitable photosensitive panchromatic emulsions and the required expensive and cumbersome recording setups usually located in dedicated holographic laboratories outside museums. The HiH has developed the necessary transportable equipment and processes for the in-situ recording of color Denisyuk-type holograms of artifacts and their subsequent optimized display. Key characteristics of the color holograms recorded and displayed with the HiH systems are:

1. High degree of color rendition
2. Realistic object’s surface texture and optical properties rendition
3. Continuous full parallax (vertical and horizontal)
4. 180 degrees field of view
5. Realistic specular reflections
6. Realistic shadows
7. 1:1 spatial reconstruction

Although characteristics from (2) to (7) in the above list are more or less inherent in Denisyuk-type holograms, the degree of faithful colors rendition is highly dependent on the correct selection of laser wavelengths, well balanced intensity and energy dosage per color, chemical processing, drying procedure and spectral characteristics of the illuminating device during reconstruction of the hologram.

The exposure levels, chemical processing and the drying procedure for a specific type of holographic plates are typically optimized by exposing a batch of test plates. Assuming that there are no distortions caused by the developing and drying processes, a perfect reconstruction can be achieved if a color reflection hologram is illuminated by the same lasers and at the same geometry used during recording. It is desirable, due to security and cost reasons, to use illuminating sources other than lasers for the reconstruction of Denisyuk-type holograms while ensuring a high degree of color rendition and image sharpness. For years, the preferred illumination sources for monochromatic Denisyuk-type holograms have been common tungsten halogen spots but in the case of color Denisyuk holograms, only a small part of their wide emission spectrum contributes to image formation, while the rest and larger part is absorbed or scattered lowering the image’s contrast. A good approximation to the perfect reconstruction can be achieved using illuminating devices such as the HoLoFoS^TM^, which incorporates intensity mixing of color LEDs with dominant wavelengths that match the wavelengths of the lasers used for hologram recording and small emitter sizes and narrow bandwidths.

The HiH transportable Z3^RGB^ color holography system and the HoLoFoS^TM^ illuminators have been successfully used to produce OptoClones^TM^ for documenting artifacts in Museums in Greece and Russia. In the BXM case, it was the first time that color holograms of cultural artifacts were recorded with a mobile holography system in situ, in a museum, and subsequently displayed in place of the original artifacts while the originals were on loan.

On another occasion involving State Treasures from the Diamond Fund of Russia, the recorded OptoClones^TM^ exhibited inside the Moscow Kremlin were highly appraised by officials and international experts as well as the general public, allowing reasonable optimism for the prospects of Display Holography for museums.

Clearly, the epitome of such remarks are the comments of the Premier Minister of the Russian Federation Dmitry Medvedev publicly declaring on-camera that “these OptoClones must travel within the country in order to be witnessed by all those who have no physical access to the original objects at the Kremlin Diamond Fund” [12].

We believe that true-color holograms, as they actually are the most advanced three dimensional images obtainable with current technologies, will become valuable tools for the preservation, documentation and diffusion of cultural heritage.

## Figures and Tables

**Table 1:** Typical developer and bleach solutions used.

Holographic Plate	Developer	Bleach
Ultimate plates by Ultimate Holography (FR)	Ultimate developer (supplied by Ultimate Holography)	Ultimate bleach (supplied by Ultimate Holography)
BBpan plates supplied to HiH by Color Holographic (UK) under exclusive non-commercial license	**Part A** 20 gr Catechol 10 gr Ascorbic Acid 10 gr Sodium Sulfite 50 gr Urea Dissolve in 1L of distilled water	30 gr Ferric Sodium EDTA 30 gr Potassium Bromide 30 gr Sodium Bisulfate Dissolve in 1 L of distilled water.
	**Part B** 60 gr anhydrous Sodium Dissolve in 1L of distilled water. Mix equal volumes of Part A and Part B just before use.	

**Table 2:** Byzantine & Christian Museum of Athens artifacts recorded as OptoClones^TM^.

OptoClone^TM^ Plate Number	Recorded Artifacts	BXM Catalogue Number
OptoClone-BXM1	Pair of earrings, 4th c. AD	BXM 175 a–b
	Pair of earrings, 4th c. AD	BXM 177 a–b
OptoClone-BXM2	Golden bracelet, 7th c. AD	BXM 888
	Amulet with chain, 7th c. AD	BXM 847
	Golden belt, 7th c. AD	BXM 879
OptoClone-BXM3	Silver ladle, 7th c. AD	BXM 899
OptoClone-BXM4	Silver plate, 7th c. AD	BXM 894
OptoClone-BXM5	Silver candle holder, 7th c. AD	BXM 900
OptoClone-BXM6	Earrings, 4th c. AD	BXM 1181 a–b
OptoClone-BXM7	Eulogy of St. Mamas 6th c.AD	ΒΧΜ 285

**Table 3:** Recorded Fabergé Eggs as OptoClones^TM^.

Imperial Easter Egg	Artist	Gift to
*Hen Egg* 1885	E. Kollin	Empress Maria Fyodorovna from Emperor Alexander III
*Renaissance Easter Egg* 1894	M. Perkhin	Empress Maria Fyodorovna from Emperor Alexander III
*Rosebud Egg* 1895	M. Perkhin	Empress Alexandra Fyodorovna from Emperor Nicholas II
*Coronation Easter Egg* 1897	M. Perkhin G. Stein	Empress Alexandra Fyodorovna from Emperor Nicholas II
*Lilies of the Valley Easter Egg* 1898	M. Perkhin G. Stein	Dowager Empress Maria Fyodorovna from Emperor Nicholas II
*Cockerel Easter Egg-Clock* 1900	M. Perkhin	Dowager Empress Maria Fyodorovna from Emperor Nicholas II
*Bay Tree Easter Egg* 1911	House of Fabergé	Dowager Empress Maria Fyodorovna from Emperor Nicholas II
*Fifteenth Anniversary Easter Egg* 1911	H. Wigström V. Zuev	Empress Alexandra Fyodorovna from Emperor Nicholas II
*Order of St. George Easter Egg* 1916	House of Fabergé	Dowager Empress Maria Fyodorovna from Emperor Nicholas II
Imperial Quality Eggs	Artist	Gift to
*Kelch Hen Egg* 1898	M. Perkhin	Presented to A. Kelch to his wife Barbara Kelch on the Easter of 1898
*Duchess of Marlborough Egg Clock* 1902	M. Perkhin	The Duchess brought this egg back to England as a souvenir from Russia
*Chanticleer Egg Clock* 1904	M. Perkhin	Presented to A. Kelch to his wife Barbara Kelch on the Easter of 1904
Not an Egg	Artist	
*Desk Timepiece with a Globe* 1908–1917	H. Wigström	

**Table 4:** Gokhran Diamond Fund objects recorded as OtptoClones^TM^.

Title	Catalogue Number	OptoClone^TM^ Dimensions
*Imperial Whistle Cum Rattle*	AF-49	40 × 30 cm
*Diadem ‘Elegeia’*	No 11_2	40 × 30 cm
*Order of St.Catherine*	INV-18750-1-4	40 × 30 cm
*Troika*	INV-350	40 × 30 cm
*Esclavage (Bow)*	AF-47	40 × 30 cm
*Shepherd*	INV-1782	30 × 40 cm
*Order of St. Alexander Nevsky*	AF-63	40 × 30 cm
*Perfume Bottle Faberge*	INV-298	20 × 25 cm
*Faberge Scoop*	INV-1201	30 × 20 cm
*Caesar’s Ruby (Pink Tourmaline Pendant)*	AF-12	20 × 25 cm
*Bell*	INV-1177	20 × 25 cm
